# High Concentration of Glucose Increases Reactive Oxygen Species Generation and Apoptosis Induced by Endoplasmic Reticulum Stress Pathway in Rabbit Corneal Epithelial Cells

**DOI:** 10.1155/2018/8234906

**Published:** 2018-07-08

**Authors:** Jiajun Lu, Minjie Sheng, Panpan Yao, Chaochao Ran, Hao Liu, Li Chen, Rui Liu, Bing Li

**Affiliations:** Department of Ophthalmology, Yangpu Hospital, Tongji University School of Medicine, Shanghai 200090, China

## Abstract

**Objective:**

To investigate the effect of high concentration of glucose on reactive oxygen species (ROS) production in rabbit corneal epithelial cells (RECEs) and explore whether the increased ROS initiates the apoptosis process of RECEs through oxidative stress and endoplasmic reticulum (ER) stress pathway.

**Methods:**

RECEs were treated by different concentrations of glucose for a while, and then the production of ROS was detected by flow cytometry. The expressions of PERK, p-PERK, Akt, p-Akt, and CHOP were determined by western blot, and the cell viability was measured by Cell Counting Kit-8 (CCK-8). Flow cytometry was used to detect the early apoptosis rate. Meanwhile, the effects of *N*-acetyl-L-cysteine (NAC), an active oxygen inhibitor, on the experimental results were observed.

**Results:**

Compared with the normal glucose concentration group, the fluorescence intensity of ROS in the high concentration (1 mM glucose) of glucose group was significantly increased (*P* < 0.05). NAC-inhibited ROS production was induced by high concentration of glucose (*P* < 0.05).Western blot demonstrated that the expressions of the p-PERK and CHOP increased significantly (*P* < 0.05), the p-Akt expression decreased (*P* < 0.05), and the PERK and Akt expressions did not change significantly in the high concentration of glucose group compared to the normal concentration group. CCK-8 results revealed that compared with the normal concentration of glucose group, the cell activity of the high concentration of glucose group decreased. For the cells in the high concentration of glucose group, the cell survival rate of NAC-treated cells was higher than that of untreated (*P* < 0.05). The flow cytometry results indicated that the early apoptosis rate of the cells in the high concentration of glucose group increased in contrast with that in the normal concentration of glucose group (*P* < 0.05). Treating the cells in the high concentration of glucose group with NAC could reduce the cell apoptosis resulted from high glucose (*P* < 0.05).

**Conclusions:**

High concentration of glucose may induce the formation of ROS which leads to oxidative stress and ER stress in RECEs and even leads to cell apoptosis. The reactive oxygen inhibitor, NAC, can play a protective character in the high concentration of glucose environment. These results might provide theoretical basis for the study of the diabetes-related dry eye.

## 1. Introduction

Diabetes is a group of metabolic diseases characterized by an increase in chronic blood glucose level caused by defects in insulin secretion and function. Diabetes has become one of the most common chronic diseases in developed countries such as Europe and the United States, and China is also confronted with the same problem. According to statistics, there are about 109 million adult diabetic patients in China, accounting for 10.9% of the total population [1].

Diabetes could cause numerous complications; among them, dry eye is one of the most common clinical symptoms [2]. Several abnormalities induced by diabetes, such as decrease of tear secretion, tear composition changes, decreased corneal sensitivity, and delayed corneal epithelial regeneration, would cause ocular surface damage. Experiments uncovered that the concentration of the tear glucose in diabetic patients was 5 times higher than that in normal people, and the concentration of glucose in tears was significantly correlated with blood glucose [3]. Tear osmotic pressure can be significantly increased due to the high tear glucose, which increases with the duration of diabetes [4]. Glucose can be converted to deoxyglucosone in the high glucose condition and would produce a good deal of ROS accompanied by the self-oxidation [5]. Endoplasmic reticulum (ER) is an organelle and the place of proteins folded and assembled in eukaryotic cells. ROS can change the channel function of the ER and the buffer of chaperones, affecting the balance of calcium ions. In order to maintain intracellular homeostasis, an unfolded protein response (UPR) is initiated to protect the endoplasmic reticulum [6]. UPR is mainly mediated by three kinds of signal transducers, including activating transcription factor 6 (ATF6), type-1 ER transmembrane protein kinase (IRE-1), and double-stranded RNA-dependent protein kinase-like ER kinase (PERK) [7]. With the deterioration of the situation, UPR cannot maintain the balance of proteins and calcium within ER, which would activate the apoptotic pathway [8]. On the basis of these backgrounds, we established a model in vitro to explore the circumstance of ROS production in high concentration of glucose environment and the relevant involvement of ER stress in the rabbit corneal epithelial cells (RECEs) apoptosis. We further evaluated the effects of the reactive oxygen inhibitor, *N*-acetyl-L-cysteine (NAC), on the cellular protection and tried to elucidate the underlying pathological mechanism of diabetes-related dry eye.

## 2. Materials and Methods

### 2.1. Ethics Statement

The experimental protocol was approved by the Ethics Committee of the Yangpu Hospital, Tongji University Medical School. All animal procedures and experiments were approved by the Animal Care and Use Committee in Tongji University Medicine School. All animals were cared according to the Association for Research in Vision and Ophthalmology statement for using animals in ophthalmic and vision research. In addition, every surgery was performed under sodium pentobarbital anesthesia, and all efforts were made to minimize suffering.

### 2.2. Animals and Cell Culture

50 male White New Zealand rabbits with a mean weight of 2.25 ± 0.25 kg were obtained from Central Lab Tongji University Laboratory Animal Center (Shanghai, China). All the rabbits with 100 eyes were divided into five groups: OPC group, NCG group, HCG group, NCG + NAC group, and HCG + NAC group, each group with 20 eyes. RECEs were cultured and identified according to the experimental method described previously [9]. Generation 1 cells were used for subsequent experiments. RECEs were washed three times with PBS (Gibco, Grand Island, NY, USA) when they were confluent reaching 80% and maintained in serum-free keratinocyte medium (ScienCell Research Laboratories, San Diego, CA, USA) containing 100 U/ml penicillin (Gibco, Grand Island, NY, USA) and 100 mg/ml streptomycin (Gibco, Grand Island, NY, USA) 24 h. All experiments were performed in triplicate.

### 2.3. Cell Grouping and Treatment

RECEs were seeded at 4 × 10^5^/well into a 6-well plate and maintained in serum-free keratinocyte medium containing 100 U/ml penicillin and 100 mg/ml streptomycin 24 h. The cells were divided into six groups (Table 1).

### 2.4. Detection of ROS

After 24 h treatment, the medium was removed and the cells were washed with PBS three times, and then the experimenter added 10 *μ*M dichlorodihydrofluorescein diacetate (DCFH-DA, Beyotime, Shanghai, China) protected from light and placed at 37°C for 30 min. After 0.05% trypsin incubation at 37°C for 5 min, serum-containing medium was added to terminate the digestion. The cell suspension was transferred into a flow test tube and centrifuged at 800 rpm for 3 min. The supernatant was removed and 1 ml of PBS was added to each tube and centrifuged at 800 rpm for 3 min. Afterwards, the supernatant was removed and 500 *μ*l of PBS was added to each tube, and finally, ROS was detected in accordance with FITC fluorescence detection conditions for flow cytometry (BD FACSCanto II, Becton, Dickinson and Company, Franklin Lakes, NJ, USA).

### 2.5. Detection of Cell Apoptosis

The digestive cells were transferred into a flow test tube and resuspended with the binding buffer after washing three times. Cell apoptosis was detected in accordance the manufacturer's instructions of the Annexin V-fluorescein isothiocyanate/propidium iodide apoptosis kit (Annexin V-FITC/PI, Invitrogen, Carlsbad, CA, USA). Briefly, 5 *μ*l Annexin V was added, gently mixed, and then placed at room temperature for 15 min protected from light. And then, 5 *μ*l PI was added, gently mixed, and placed at room temperature for 5 min protected from light. In the end, cell apoptosis was detected within 1 hour by flow cytometry.

### 2.6. Detection of Cell Proliferation

After 24 h treatment, the medium was removed and cells were washed with PBS three times; 10 *μ*l CCK-8 (Dojindo Laboratories, Minato-ku, Tokyo, Japan) was added and placed at 37°C for 4 h protected from light. The absorbance was detected at 450 nm.

### 2.7. Western Blot Assay

RECEs were washed with PBS three times. 150 *μ*l of protein cleavage fluid was added (Beyotime, Shanghai, China) each and lysed on ice for 5 min. The cellular protein was scraped with a cell scraper, transferred to an EP tube, and then centrifuged at 4°C at 14000 rpm for 10 min. The supernatants were tested for protein concentration using the BCA method (Thermo Fisher Scientific, Waltham, MA, USA). 5 × SDS loading buffer was added to total protein, mixed well, and then heated to 100°C for denaturing. The protein was transferred to the PVDF transfer membrane (Millipore Corporation, Temecula, CA, USA) after separation by 10% SDS polyacrylamide gel. The membranes were blocked in 5% nonfat milk for 1 h and incubated with primary antibody in 1 : 500 dilution overnight at 4°C. Primary antibodies against Akt (Sigma-Aldrich, Oakville, Ontario, Canada), p-Akt (Cell Signaling Technology, Danvers, MA, USA), PERK (Cell Signaling Technology, Danvers, MA, USA), p-PERK (Millipore Corporation, Temecula, CA, USA), CHOP (Abcam, Cambridge, MA, USA), and *β*-actin (Cell Signaling Technology, Danvers, MA, USA) were used in this study. The membrane was rinsed three times with TBST for 10 minutes each and incubated with horseradish peroxidase conjugated secondary antibody against rabbit IgG (Dako, Glostrup, Denmark) in a 1 : 1000 dilution for 1 h. The membrane was rinsed three times with TBST for 10 minutes each and exposed by SuperSignal West Pico Chemiluminescent Substrate (PIERCE) for detecting the blots. The density of the bands was analyzed, and the expression of *β*-actin was used as internal control.

### 2.8. Statistical Analysis

All results were expressed as mean ± standard deviation (SD) unless indicated otherwise notified. Statistical analysis was performed with Statistical Product and Service Solutions 22.0 for Windows (SPSS 22.0 Inc., Chicago, IL, USA). ANOVA with multiple comparisons was used between groups. Differences at *P* < 0.05 were considered statistically significant.

## 3. Results

### 3.1. ROS Generation Detection by Flow Cytometry

**Figure 1:**
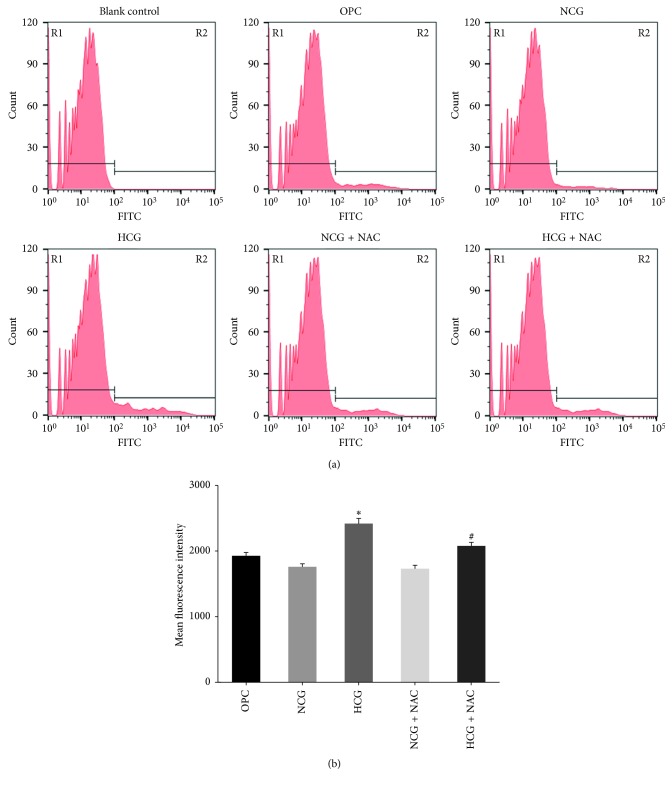
ROS generation detected by flow cytometry (a). In the blank control group, only a negative peak was detected, and positive peaks detected in other groups indicate DCF fluorescence expression. In the HCG group, the fluorescence intensity was significantly stronger than in the OPC group and the NCG group (^*∗*^*P* < 0.05 versus OPC and ^*∗*^*P* < 0.05 versus NCG). In the HCG + NAC group, the fluorescence intensity was weaker than in the HCG group (^#^*P* < 0.05 versus HCG). Statistical analysis (b) was represented as mean ± SD (^*∗*^*P* < 0.05 and ^#^*P* < 0.05).

Cell fluorescence was detected by flow cytometry. DCFH-DA has no fluorescence itself, which can freely penetrate the cell membrane into the cell and be hydrolyzed by esterase to dichlorodihydrofluorescein (DCFH). The intracellular reactive oxygen species can oxidize the nonfluorescent DCFH to produce a green fluorescent substance 2ʹ,7ʹ-dichlorofluorescein (DCF), and its fluorescence intensity is proportional to the level of intracellular reactive oxygen species. Under the FITC fluorescence condition, a negative peak was detected only in the blank control group (Figure 1(a)), while positive peak could be detected in the other five experimental groups. In the HCG group, the fluorescence intensity was significantly stronger than in the OPC group and the NCG group. In the HCG + NAC group, the fluorescence intensity was weaker than in the HCG group, and the positive peak left-deviated. Compared with OPC group and NCG group, the fluorescence intensity of ROS in the HCG group increased significantly (Figure 1(b), *P* < 0.05). Compared with HCG group, the fluorescence intensity of ROS in the HCG + NAC group decreased significantly (*P* < 0.05).

### 3.2. Cell Apoptosis Detection by Flow Cytometry

**Figure 2:**
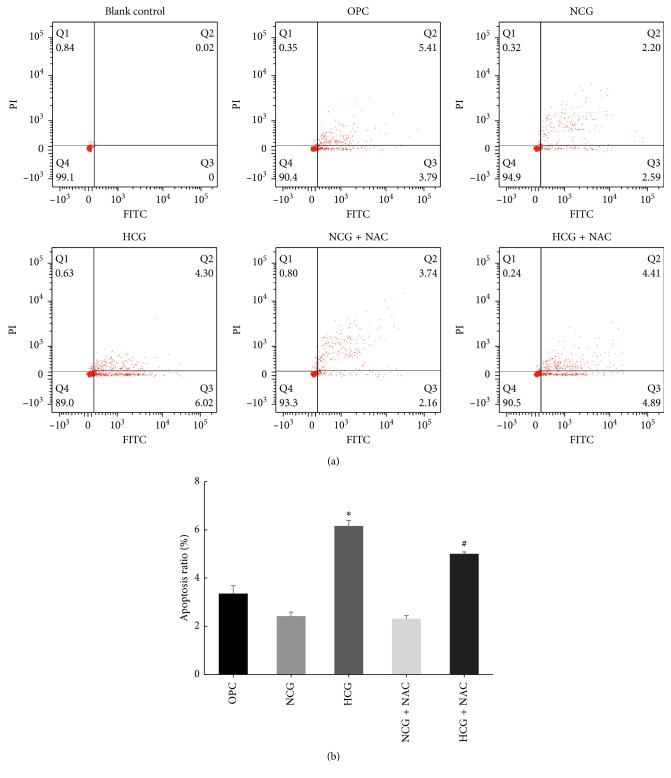
Cell apoptosis detected by flow cytometry (a). The percentage of apoptotic cells in the HCG group was significantly higher compared to those in the OPC group and the NCG group (^*∗*^*P* < 0.05 versus OPC and ^*∗*^*P* < 0.05 versus NCG), but it was significantly decreased in the HCG + NAC group (^#^*P* < 0.05 versus HCG). Statistical analysis (b) was represented as mean ± SD (^*∗*^*P* < 0.05 and ^#^*P* < 0.05).

Compared with OPC group (Figure 2(a)) and NCG group, the percentage of early apoptotic cells in the HCG group increased significantly (*P* < 0.05). Compared with HCG group, the percentage of early apoptotic cells in the HCG + NAC group decreased significantly (*P* < 0.05). There was no significant difference in the percentage of early apoptotic cells between the NCG group and the NCG + NAC group (*P* > 0.05). Statistical analysis was represented as mean ± SD in Figure 2(b) (*P* < 0.05).

### 3.3. Cell Proliferation Assay

**Figure 3:**
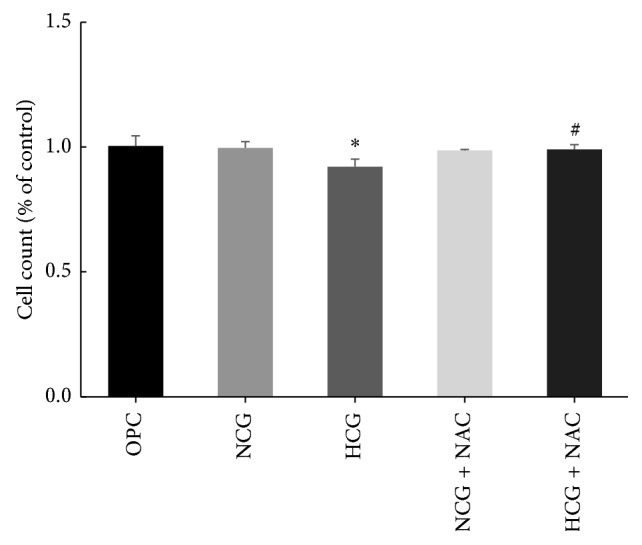
Cell proliferation assay. Statistical analysis was represented as mean ± SD (^*∗*^*P* < 0.05 versus OPC and ^#^*P* < 0.05 versus HCG).

Cells' viability after different treatments was detected by CCK-8 assay. Compared with OPC group and NCG group, cell viability in the HCG group decreased significantly (*P* < 0.05). Compared to the HCG group, cell viability in the HCG + NAC group increased significantly (*P* < 0.05). There was no obvious difference in cell viability between the NCG group and the NCG + NAC group (*P* > 0.05) (Figure 3).

### 3.4. Protein Detection by Western Blot Assay

**Figure 4:**
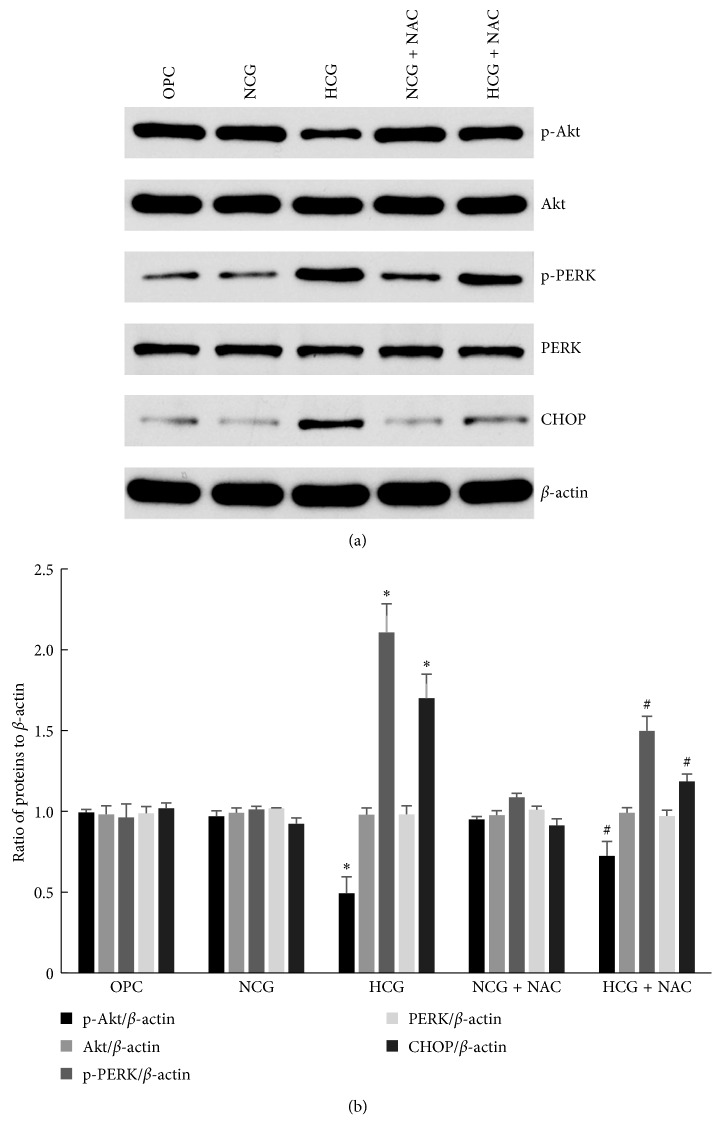
The expressions of ER stress-related molecules in RECEs. The expressions of Akt, p-Akt, PERK, p-PERK, CHOP, and *β*-actin were detected by western blot assay (a). The expressions of p-PERK and CHOP in the HCG group increased significantly than those in the OPC group and the NCG group, while p-Akt expression decreased significantly (^*∗*^*P* < 0.05 versus OPC and ^*∗*^*P* < 0.05 versus NCG). The expressions of p-PERK and CHOP in the HCG + NAC group decreased significantly than that in the HCG group, while p-Akt expression increased significantly (^#^*P* < 0.05 versus HCG). Statistical analysis (b) was represented as mean ± SD (^*∗*^*P* < 0.05 and ^#^*P* < 0.05).

Compared with OPC group and NCG group, the expressions of p-PERK and CHOP in RECEs increased significantly (*P* < 0.05), but p-Akt expression decreased significantly (*P* < 0.05) in the HCG group. Compared with the HCG group, the expressions of p-PERK and CHOP were decreased significantly (*P* < 0.05), but p-Akt expression increased significantly (*P* < 0.05) in the HCG + NAC group (Figure 4).

## 4. Discussion

For diabetic patients, metabolic disorders cause abnormal secretion and composition of tears and unstable tear film, which leads to a series of ocular disorders. Diabetes-related dry eye can lead to serious ocular surface lesions. The main clinical features include persistent corneal epithelial defects, delayed epithelial regeneration after ocular surgery, and even repeated exfoliation [10, 11]. In patients with long course of diabetes, sensitivity of corneal nerve decreases, tactile threshold increases, corneal nerve fibers narrow, branches decrease, and corneal infection and even corneal ulcer can occur [12]. It is difficult to reduce the symptoms of diabetic dry eye completely, especially the problem of persistent corneal epithelial damage. Therefore, finding out the pathogenesis of diabetes-related dry eye is very crucial.

In the previous study, the tear film rupture time and tear secretion are measured in diabetic patients, and it is found that the tear film breakage time and Schirmer value in diabetic patients are significantly lower than those in the control group; the longer the duration of diabetes, the more significant the difference is. These results uncover that the duration and the stability of the tear film in diabetic patients is poor, and thus the dry eye is serious [13]. Our previous clinical study and tear proteomics analysis confirmed that compared with normal population, diabetic patients had obvious dry eye symptoms, tear secretion decreased, tear film stability decreased, and corneal epithelial cells damaged [14].

Oxygen can form some free radicals in the normal metabolism of organisms, including superoxide anion (O_2_^−^), hydrogen peroxide (H_2_O_2_), and hydroxyl radical (OH), which are collectively referred to as reactive oxygen species (ROS). Oxidative stress refers a serious state of that stimulated by a variety of pathological factors, including production of too much of ROS, decreased antioxidant capacity, and broken normal dynamic equilibrium recovery capacity of oxygen-like body. These cause oxidative damage to the biological macromolecules, such as proteins, lipids, and nucleic acids, and thus interfere with normal life activities and form serious stress states [15]. Under normal physiological conditions, ROS acts a role in maintaining the signal transduction and normal function of cells. Endogenous antioxidant stress response systems include enzymes such as superoxide dismutase, catalase, glutathione peroxidase, and nonenzymes, such as vitamins that inhibit the accumulation of ROS. The exogenous factors, such as exogenous oxidants, radiation, and environmental or endogenous factors, such as mitochondrial respiratory chain reaction, glucose metabolism, and amino acid metabolism, can cause excessive accumulation of ROS [16]. If the cell's antioxidant stress is inhibited at the same time, the broken ROS balance will further activate cell mitochondrial damage, ER stress-related signal transduction pathway. The production of ROS can cause ER stress under various conditions [17, 18].

In this experiment, RECEs were exposed to different concentrations of high glucose to confirm that RECEs could produce more ROS under the high concentration of glucose than that under normal, while it also produced more ROS than the high concentration of glucose osmotic pressure group. Under oxidative stress, the sensitivity of cells to stress was increased. In dry eye animal models, when the production of ROS in the corneal epithelium increases beyond its compensatory capacity, it further activates the relevant cytokines and destroys the regenerative capacity of the corneal epithelial cell layer [19]. These results reveal that the increase of ROS in the cornea is closely related to the pathology of corneal epithelial injury. The results of the present study might hint that high concentration of glucose can lead to corneal damage by inducing the production of ROS in RECEs.

In the early stage of ERS, the unfolded protein response (UPR) is activated because the protein biosynthesis reduces and the ability of ER degradation enhances so as to maintain homeostasis [6]. In the event that cell injury has not been alleviated or even further enhanced, the apoptotic signaling pathway is activated because the UPR cannot maintain the balance of protein and calcium in the ER [8]. It induces eukaryotic translation initiation factor 2*α* (eIF2*α*) expression primarily through activated PERK, which leads to enhanced expression of the proapoptotic transcription factor CHOP, inducing apoptosis [20]. Our previous study [9] showed that high osmotic pressure interferes make RECEs produce ROS, and it can serve as an upstream signal of c-Jun N-terminal kinase (JNK) signaling pathway to promote JNK phosphorylation. JNK signaling pathway activation can promote the activation of preinflammatory factor nuclear factor kappaB (NF-ΚB) and then promote the expression of inflammatory cytokines interleukin-1*β* (IL-1*β*) and tumor necrosis factor alpha (TNF-*α*), leading to the inflammation of RECEs. At the same time, the increased production of ROS in RECEs can induce the apoptosis by activating the JNK signaling pathway of the apoptosis system cluster of differentiation 95 (CD95)/CD95 ligand (CD95L). In the present study, the expressions of p-PERK and CHOP increased and p-Akt expression decreased, while PERK and Akt expressions did not change significantly in the high concentration of glucose group compared with that in the normal group.

PERK is an upstream signaling molecule of CHOP, and p-PERK can activate the expression of downstream CHOP. CHOP, as a specific transcription factor of ER stress, is considered as a marker of ER stress. Under normal conditions, the expression of CHOP is quite low, while under the ER stress, the expression of CHOP would be significantly increased [21, 22]. The Akt signaling pathway plays a key regulatory role in various biological processes, such as cell metabolism, proliferation, and survival. Meanwhile, it also plays a crucial protective character in ER stress [23–26]. ER stress can cause a rapid decline in Akt phosphorylation, and the PERK-CHOP-Akt pathway plays an important role in this process [27]. In this experiment, we found that the expression of p-PERK and CHOP increased in the HCG group. CHOP could negatively regulate the activation of Akt during the process of high concentration of glucose-induced RECEs apoptosis. Since CHOP is a sign of ER stress, the results indicated that it was accompanied with ER stress in the HCG group.

Abnormal cell apoptosis is presented in the ocular surface tissues of dry eye patients; the proapoptotic factors in the tear and the ocular surface, for instance, Fas (factor-associated suicide)/FasL (fas ligand) and Bax (B-cell lymphoma-2-associated X protein) along with inflammatory factors such as interleukin-1 (IL-1), tumor necrosis factor alpha (TNF-*α*), and interleukin 6 (IL-6) would increase its expressions and activate apoptotic pathway. It is explicit that there exists apoptosis in dry eyes. Nevertheless, we cannot simply discuss the causal relationship between them. The connection between the two can be a vicious cycle that promotes each other, and both sides are mutually causative. When the accumulation of apoptotic cells reaches a certain quantity, it would inevitably affect the organ function.

Flow cytometry uncovered that the early apoptosis rate of the HCG group was significantly higher than that in the NCG group. In addition, the early apoptotic rate of the HCG group with antioxidant NAC was obviously lower than that of the HCG group. Analysis of cell viability demonstrated that the RECEs' viability decreased remarkably with the increase of high concentration of glucose. NAC is a cysteine acetyl derivative with free radical scavenging activity and a precursor of reduced glutathione in human body [28]. As a commonly used antioxidant, it plays an important role in the treatment of many diseases such as heart, kidney, respiration, liver, and mental illness. Our study found that NAC could reduce the production of ROS induced by high concentration of glucose in RECEs because the fluorescence intensity of the HCG group was significantly decreased by adding NAC. P-PERK and CHOP expressions were downregulated, and p-Akt expression was upregulated after NAC was added to the HCG group RECEs. The results of flow cytometry displayed that the early apoptosis rate of the HCG group was significantly lower and the cell viability was significantly increased by adding the ROS inhibitor NAC. The outcomes showed that NAC could inhibit the generation of ROS and further reduce the intensity of cellular ER stress. It was confirmed that ROS generation is an upstream event of ER stress and the protective effect of NAC in the high glucose environment.

## 5. Conclusions

In summary, our results disclosed that ROS production in high concentration of glucose-induced RECEs triggers apoptosis by activating ER stress pathways. This might explain why diabetics are more prone to dry eye symptoms. Antioxidant NAC could protect RECEs under high concentration of glucose conditions. This work may provide new ideas for the clinical treatment of dry eye caused by diabetes.

## Figures and Tables

**Table 1:** The rabbit corneal epithelial cells grouping and treatment.

Number	Group	Treatment
1	Blank control	—
2	OPC	0.5 mM sodium chloride
3	NCG	0.12 mM glucose
4	HCG	1 mM glucose
5	NCG + NAC	0.12 mM glucose + NAC
6	HCG + NAC	1 mM glucose + NAC

OPC: osmotic pressure control; NCG: normal concentration of glucose; HCG: high concentration of glucose; NAC: *N*-acetyl-L-cysteine (Sigma-Aldrich, Oakville, Ontario, Canada).

## Data Availability

The data used to support the findings of this study are available from the corresponding author upon request.

## References

[1] Wang L., Gao P., Zhang M. (2017). Prevalence and ethnic pattern of diabetes and prediabetes in China in 2013. *Journal of the American Medical Association*.

[2] Kaiserman I., Kaiserman N., Nakar S., Vinker S. (2005). Dry eye in diabetic patients. *American Journal of Ophthalmology*.

[3] Baca J. T., Taormina C. R., Feingold E., Finegold D. N., Grabowski J. J., Asher S. A. (2007). Mass spectral determination of fasting tear glucose concentrations in nondiabetic volunteers. *Clinical Chemistry*.

[4] Sagdik H. M., Tetikoğlu M., Uçar F., Uğurbaş S. C., Ugurbas S. H. (2013). Tear film osmolarity in patients with diabetes mellitus. *Ophthalmic Research*.

[5] Paravicini T. M., Touyz R. M. (2008). NADPH oxidases, reactive oxygen species, and hypertension: clinical implications and therapeutic possibilities. *Diabetes Care*.

[6] Chistiakov D. A., Sobenin I. A., Orekhov A. N., Bobryshev Y. V. (2014). Role of endoplasmic reticulum stress in atherosclerosis and diabetic macrovascular complications. *Biomed Research International*.

[7] Dai M. X., Zheng X.-H., Yu J. (2014). The impact of intermittent and repetitive cold stress exposure on endoplasmic reticulum stress and instability of atherosclerotic plaques. *Cellular Physiology and Biochemistry*.

[8] Tadic V., Prell T., Lautenschlaeger J., Grosskreutz J. (2014). The ER mitochondria calcium cycle and ER stress response as therapeutic targets in amyotrophic lateral sclerosis. *Frontiers in Cellular Neuroscience*.

[9] Chen Y., Li M., Li B., Wang W., Lin A., Sheng M. (2013). Effect of reactive oxygen species generation in rabbit corneal epithelial cells on inflammatory and apoptotic signaling pathways in the presence of high osmotic pressure. *PLoS One*.

[10] Xu K., Yu F. S. (2011). Impaired epithelial wound healing and EGFR signaling pathways in the corneas of diabetic rats. *Investigative Opthalmology and Visual Science*.

[11] Xu K. P., Li Y., Ljubimov A. V., Yu F.-S. X. (2009). High glucose suppresses epidermal growth factor receptor/phosphatidylinositol 3-kinase/Akt signaling pathway and attenuates corneal epithelial wound healing. *Diabetes*.

[12] Dogru M., Katakami C., Inoue M. (2001). Tear function and ocular surface changes in noninsulin-dependent diabetes mellitus. *Ophthalmology*.

[13] Rocha E. M., Mantelli F., Nominato L. F., Bonini S. (2013). Hormones and dry eye syndrome: an update on what we do and don’t know. *Current Opinion in Ophthalmology*.

[14] Li B., Sheng M., Xie L. (2014). Tear proteomic analysis of patients with type 2 diabetes and dry eye syndrome by two-dimensional nano-liquid chromatography coupled with tandem mass spectrometry. *Investigative Opthalmology and Visual Science*.

[15] Barbieri E., Sestili P. (2012). Reactive oxygen species in skeletal muscle signaling. *Journal of Signal Transduction*.

[16] Perjes A., Kubin A. M., Kónyi A. (2012). Physiological regulation of cardiac contractility by endogenous reactive oxygen species. *Acta Physiologica*.

[17] Pierre N., Barbé C., Gilson H., Deldicque L., Raymackers J.-M., Francaux M. (2014). Activation of ER stress by hydrogen peroxide in C2C12 myotubes. *Biochemical and Biophysical Research Communications*.

[18] Jun A. S., Meng H., Ramanan N. (2012). An alpha 2 collagen VIII transgenic knock-in mouse model of Fuchs endothelial corneal dystrophy shows early endothelial cell unfolded protein response and apoptosis. *Human Molecular Genetics*.

[19] Zheng Q., Ren Y., Reinach P. S. (2015). Reactive oxygen species activated NLRP3 inflammasomes initiate inflammation in hyperosmolarity stressed human corneal epithelial cells and environment-induced dry eye patients. *Experimental Eye Research*.

[20] Duan Z., Zhao J., Fan X. (2014). The PERK-eIF2alpha signaling pathway is involved in TCDD-induced ER stress in PC12 cells. *Neurotoxicology*.

[21] Aslan M., Basaranlar G., Unal M., Ciftcioglu A., Derin N., Mutus B. (2014). Inhibition of neutral sphingomyelinase decreases elevated levels of inducible nitric oxide synthase and apoptotic cell death in ocular hypertensive rats. *Toxicology and Applied Pharmacology*.

[22] Zode G. S., Sharma A. B., Lin X. (2014). Ocular-specific ER stress reduction rescues glaucoma in murine glucocorticoid-induced glaucoma. *Journal of Clinical Investigation*.

[23] Wang Z., Wang Y., Ye J. (2015). bFGF attenuates endoplasmic reticulum stress and mitochondrial injury on myocardial ischaemia/reperfusion via activation of PI3K/Akt/ERK1/2 pathway. *Journal of Cellular and Molecular Medicine*.

[24] Zhang W., Neo S. P., Gunaratne J. (2015). Feedback regulation on PTEN/AKT pathway by the ER stress kinase PERK mediated by interaction with the Vault complex. *Cellular Signalling*.

[25] Lin M. L., Chen S.-S., Huang R.-Y. (2014). Suppression of PI3K/Akt signaling by synthetic bichalcone analog TSWU-CD4 induces ER stress- and Bax/Bak-mediated apoptosis of cancer cells. *Apoptosis*.

[26] Zhou Y., Liang X., Chang H. (2014). Ampelopsin-induced autophagy protects breast cancer cells from apoptosis through Akt-mTOR pathway via endoplasmic reticulum stress. *Cancer Science*.

[27] Martin-Perez R., Palacios C., Yerbes R. (2014). Activated ERBB2/HER2 licenses sensitivity to apoptosis upon endoplasmic reticulum stress through a PERK-dependent pathway. *Cancer Research*.

[28] Shalansky S. J., Vu T., Pate G. E., Levin A., Humphries K. H., Webb J. G. (2005). N-acetylcysteine for prevention of radiographic contrast material-induced nephropathy: is the intravenous route best?. *Pharmacotherapy*.

